# Systematic review about the screening of cannabis use during pregnancy and neonates

**DOI:** 10.1192/j.eurpsy.2021.2180

**Published:** 2021-08-13

**Authors:** A. Gonzalez-Mota, M. Covacho-Gonzalez, I. Valriberas-Herrero, C. Roncero, J. De La Iglesia-Larrad

**Affiliations:** Psychiatry, University of Salamanca Healthcare Complex. Institute of Biomedicine of Salamanca, Salamanca, Spain

**Keywords:** neonates, Screening, Cannabis, pregnancy

## Abstract

**Introduction:**

Cannabis use in pregnancy is related to developmental and mental disorders. The acknowledgement of prenatal exposure frequently depends on the mother’s report, which can often be omitted. There exists little bibliography of the different methods to detect the use of cannabis during pregnancy, with no standardized screening available.

**Objectives:**

The objective of this study is to review the available bibliography about screening of cannabis use during pregnancy and neonates and to analyze the different methods of prenatal screening being used in clinical practice.

**Methods:**

A systematic review of the methods of screening of cannabis use during pregnancy and neonates was carried out in PubMed (July 2020) in English, French and Spanish(10 years) with the keywords: screening, test, detection, analysis, urine, blood, hair, meconium.107 studies were analyzed: 52 included and 55 excluded (Figure 1.).

**Figure:**
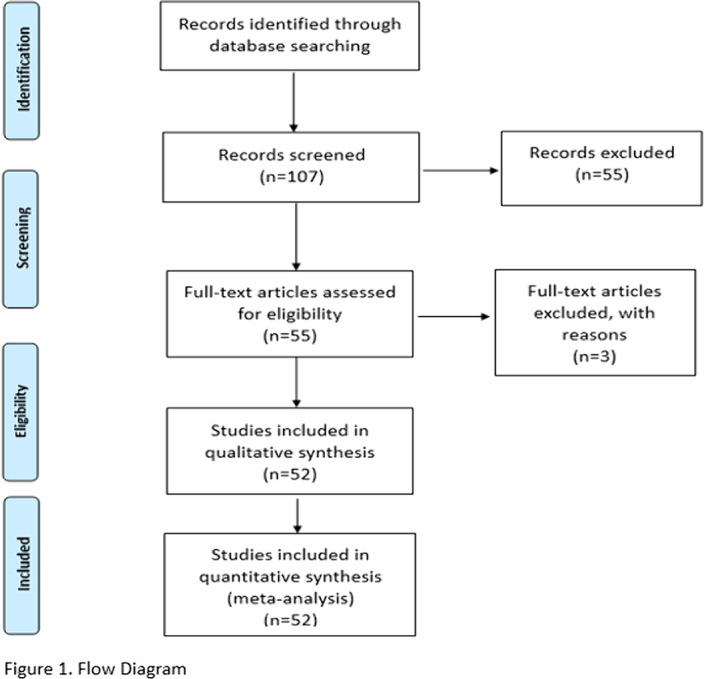

**Results:**

The studies analyzed stand out for its large heterogeneity. Self-report of pregnant women, meconium and maternal urine analysis are used the most. The type of analysis technique is not reported or chromatography mass spectrometry (GC/MS) and enzyme-linked inmunoabsorbent assay (ELISA) is used(Figure 2.). Urine seems to be the most accurate method for maternal testing. Neonatal meconium and umbilical cord tissue indicates fetal exposure during second and third trimester, neonatal hair third trimester exposure and maternal serum and hair can also be used (Figure 3.).

**Figure:**
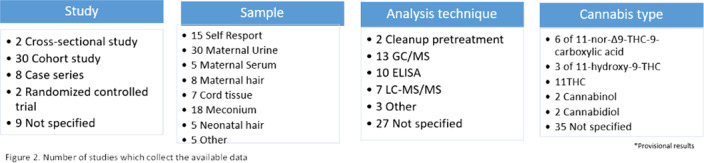

**Figure:**
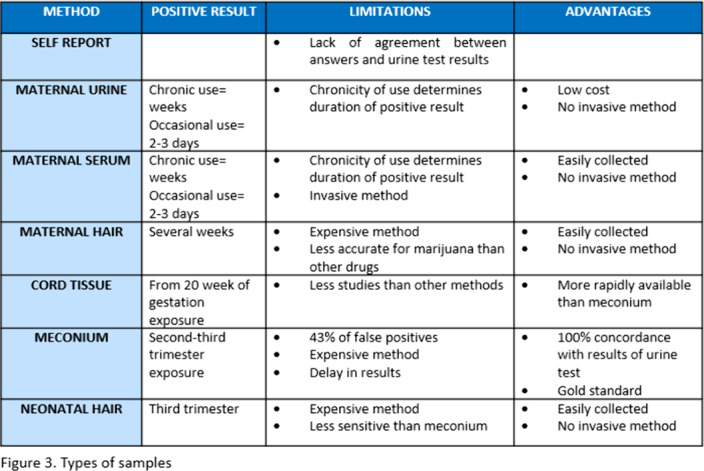

**Conclusions:**

Nowadays, the available bibliography is heterogeneous and lacks information. Consequentially, further investigation needs to be carried out in order as to establish standardized prenatal screening of cannabis during pregnancy to draw more comparable and precise conclusions.

**Disclosure:**

No significant relationships.

